# Glyphosate, pathways to modern diseases II: Celiac sprue and gluten intolerance

**DOI:** 10.2478/intox-2013-0026

**Published:** 2013-12

**Authors:** Anthony Samsel, Stephanie Seneff

**Affiliations:** 1 Independent Scientist and Consultant, Deerfield, NH 03037, USA; 2 Computer Science and Artificial Intelligence Laboratory, MIT, Cambridge, MA, USA

**Keywords:** celiac disease, gluten, glyphosate, food, cytochrome P450, deficiency

## Abstract

Celiac disease, and, more generally, gluten intolerance, is a growing problem worldwide, but especially in North America and Europe, where an estimated 5% of the population now suffers from it. Symptoms include nausea, diarrhea, skin rashes, macrocytic anemia and depression. It is a multifactorial disease associated with numerous nutritional deficiencies as well as reproductive issues and increased risk to thyroid disease, kidney failure and cancer. Here, we propose that glyphosate, the active ingredient in the herbicide, Roundup^®^, is the most important causal factor in this epidemic. Fish exposed to glyphosate develop digestive problems that are reminiscent of celiac disease. Celiac disease is associated with imbalances in gut bacteria that can be fully explained by the known effects of glyphosate on gut bacteria. Characteristics of celiac disease point to impairment in many cytochrome P450 enzymes, which are involved with detoxifying environmental toxins, activating vitamin D3, catabolizing vitamin A, and maintaining bile acid production and sulfate supplies to the gut. Glyphosate is known to inhibit cytochrome P450 enzymes. Deficiencies in iron, cobalt, molybdenum, copper and other rare metals associated with celiac disease can be attributed to glyphosate's strong ability to chelate these elements. Deficiencies in tryptophan, tyrosine, methionine and selenomethionine associated with celiac disease match glyphosate's known depletion of these amino acids. Celiac disease patients have an increased risk to non-Hodgkin's lymphoma, which has also been implicated in glyphosate exposure. Reproductive issues associated with celiac disease, such as infertility, miscarriages, and birth defects, can also be explained by glyphosate. Glyphosate residues in wheat and other crops are likely increasing recently due to the growing practice of crop desiccation just prior to the harvest. We argue that the practice of “ripening” sugar cane with glyphosate may explain the recent surge in kidney failure among agricultural workers in Central America. We conclude with a plea to governments to reconsider policies regarding the safety of glyphosate residues in foods.

## 1 Introduction

Gluten intolerance is a growing epidemic in the U.S. and, increasingly, worldwide. Celiac sprue is a more specific disorder, characterized by gluten intolerance along with autoantibodies to the protein, transglutaminase, which builds crosslinks in undigested fragments of gliadin, a major constituent of gluten (Green & Cellier, 2007). The autoantibodies are produced as an immune response to undegraded fragments of proteins in gluten. A remarkable set of symptoms develop over time in association with celiac disease, including weight loss, diarrhea, chronic fatigue, neurological disorders, anemia, nausea, skin rashes, depression, and nutrient deficiencies. Usually, but not always, a strict gluten-free diet can alleviate many of the symptoms. A key associated pathology is an inflammatory response in the upper small intestine, leading to villous atrophy, a flattening of the microvilli which impairs their ability to function in their important role in absorbing nutrients.

Some have suggested that the recent surge in celiac disease is simply due to better diagnostic tools. However, a recent study tested frozen sera obtained between 1948 and 1954 for antibodies to gluten, and compared the results with sera obtained from a matched sample from people living today (Rubio-Topia *et al.*, 2009). They identified a four-fold increase in the incidence of celiac disease in the newer cohort compared to the older one. They also determined that undiagnosed celiac disease is associated with a 4-fold increased risk of death, mostly due to increased cancer risk. They concluded that the prevalence of undiagnosed celiac disease has increased dramatically in the United States during the past 50 years.

Transglutaminases play many important roles in the body, as they form covalent crosslinks in complex proteins in connection with blood coagulation, skin-barrier formation, extracellular matrix assembly, and fertilization, endowing the substrate with protection from degradation by proteases (Lorand & Graham, 2003). They also form crosslinks in undigested fragments of gliadin derived from wheat, and sensitivity to certain of these fragments leads to the development of autoantibodies to tissue transglutaminase (Esposito *et al.*, 2002) that inhibit its activity.

Glyphosate is the active ingredient in the herbicide Roundup. It is a broad-spectrum herbicide, considered to be nearly nontoxic to humans (Williams *et al.*, 2000). However, a recent paper (Samsel & Seneff, 2013), argued that glyphosate may be a key contributor to the obesity epidemic and the autism epidemic in the United States, as well as to several other diseases and conditions, such as Alzheimer's disease, Parkinson's disease, infertility, depression, and cancer. Glyphosate suppresses 5-enolpyruvylshikimic acid-3-phosphate synthase (EPSP synthase), the rate-limiting step in the synthesis of the aromatic amino acids, tryptophan, tyrosine, and phenylalanine, in the shikimate pathway of bacteria, archaea and plants (de María *et al.*, 1996). In plants, aromatic amino acids collectively represent up to 35% of the plant dry mass (Franz, 1997). This mode of action is unique to glyphosate among all emergent herbicides. Humans do not possess this pathway, and therefore we depend upon our ingested food and our gut microbes to provide these essential nutrients. Glyphosate, patented as an antimicrobial (Monsanto Technology LLC, 2010), has been shown to disrupt gut bacteria in animals, preferentially killing beneficial forms and causing an overgrowth of pathogens. Two other properties of glyphosate also negatively impact human health – chelation of minerals such as iron and cobalt, and interference with cytochrome P450 (CYP) enzymes, which play many important roles in the body. We will have much more to say about these aspects in later sections of this paper.

**Figure 1:**
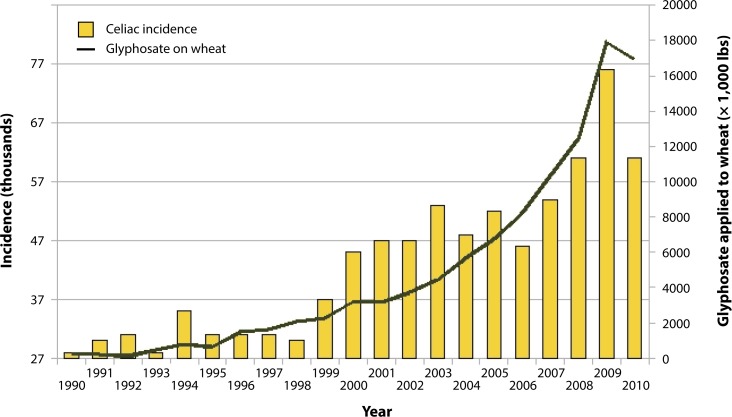
Hospital discharge diagnosis (any) of celiac disease ICD-9 579 and glyphosate applications to wheat (R=0.9759, *p≤*1.862e-06). Sources: USDA:NASS; CDC. (Figure courtesy of Nancy Swanson).

A recent study on glyphosate exposure in carnivorous fish revealed remarkable adverse effects throughout the digestive system (Senapati *et al.*, 2009). The activity of protease, lipase, and amylase were all decreased in the esophagus, stomach, and intestine of these fish following exposure to glyphosate. The authors also observed “disruption of mucosal folds and disarray of microvilli structure” in the intestinal wall, along with an exaggerated secretion of mucin throughout the alimentary tract. These features are highly reminiscent of celiac disease. Gluten peptides in wheat are hydrophobic and therefore resistant to degradation by gastric, pancreatic and intestinal proteases (Hershko & Patz, 2008). Thus, the evidence from this effect on fish suggests that glyphosate may interfere with the breakdown of complex proteins in the human stomach, leaving larger fragments of wheat in the human gut that will then trigger an autoimmune response, leading to the defects in the lining of the small intestine that are characteristic of these fish exposed to glyphosate and of celiac patients. As illustrated in Figure 1, the usage of glyphosate on wheat in the U.S. has risen sharply in the last decade, in step with the sharp rise in the incidence of Celiac disease. We explain the reasons for increased application of glyphosate to wheat in Section 13.

In the remainder of this paper, we will first show that gut dysbiosis, brought on by exposure to glyphosate, plays a crucial role in the development of celiac disease. Many CYP enzymes are impaired in association with celiac disease, and we show that glyphosate's known suppression of CYP enzyme activity in plants and animals plausibly explains this effect in humans. In Section 4, we describe the role of excess retinoic acid in celiac disease, and show how this ties also to reproductive problems. We link this to the known effects of glyphosate on retinoic acid, mediated by its suppression of CYP enzymes. Section 5 addresses cobalamin deficiency, a known pathology associated with celiac disease that leads to macrocytic anemia. We argue that this follows as a direct consequence of glyphosate's ability to chelate cobalt. Section 6 discusses in more depth the role of anemia in celiac disease, a consequence of both cobalamin and iron deficiency. Section 7 discusses molybdenum deficiency and its link to microcephaly, which is associated with celiac disease. Section 8 discusses the link between selenium deficiency and autoimmune thyroid disease. Section 9 discusses kidney disease in connection with celiac disease and glyphosate. Section 10 discusses various nutritional deficiencies associated with celiac disease, and shows how these can directly be explained by glyphosate. Section 11 discusses the link between celiac disease and certain rare cancers that have also been linked to glyphosate. Section 12 goes into an in-depth discussion of how glyphosate might promote autoantibodies to transglutaminase. Following a section which presents compelling evidence that glyphosate residues in wheat, sugar and other crops are likely increasing in recent decades, and a section discussing the increased risk to kidney failure in agricultural workers exposed to excess glyphosate occupationally, we close with a discussion section that summarizes our findings, and a conclusion which implores governments to pay more attention to the damaging consequences of the escalation in chemical warfare on weeds that characterizes current agricultural practices.

## 2 Gut bacteria

In this section, we first discuss the role of pathogens in inducing the breakdown of tight junctions in enterocytes lining the small intestinal wall. We then show that glyphosate is associated with an overgrowth of pathogens along with an inflammatory bowel disease in animal models. A parallel exists with celiac disease where the bacteria that are positively and negatively affected by glyphosate are overgrown or underrepresented respectively in association with celiac disease in humans. We also discuss how the beneficial bacteria that are negatively impacted by glyphosate can protect from celiac disease through their enzymatic activities on gluten, and point to several articles recommending treatment plans based on probiotics.

**Figure 2:**
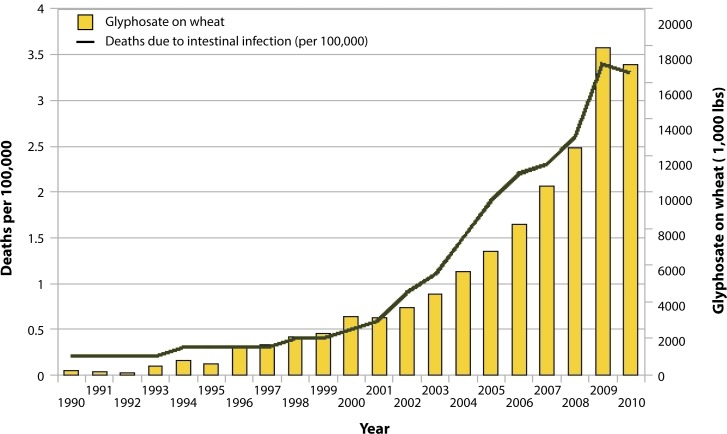
Deaths due to intestinal infections ICD A04, A09; 008, 009 with glyphosate applications to wheat (R=0.9834, *p≤*3.975e-09). Sources: USDA:NASS; CDC. (Figure courtesy of Nancy Swanson).

Pathogens, through their activation of a potent signaling molecule called zonulin, induce a breakdown of the tight junctions in cells lining the gut, leading to “leaky gut” syndrome (Fasano, 2011). Concentrations of zonulin were sharply elevated (*p<*0.000001) in subjects with celiac disease during the acute phase (Fasano *et al.*, 2000). As many as 30% of celiac patients continue to experience GI symptoms after adopting a gluten-free diet, despite optimal adherence, a condition that was attributed to bacterial overgrowth in the small intestine (Tursi *et al.*, 2003). Figure 2 shows that there is a correlation between glyphosate application to wheat and the incidence of intestinal infections.

Evidence of disruption of gut bacteria by glyphosate is available for poultry (Shehata *et al.*, 2013), cattle (Krüger *et al.*, 2013), and swine (Carman *et al.*, 2013). Glyphosate disrupts the balance of gut bacteria in poultry (Shehata *et al.*, 2013), increasing the ratio of pathogenic bacteria to other commensal microbes. Salmonella and Clostridium are highly resistant to glyphosate, whereas Enterococcus, Bifidobacteria, and Lactobacillus are especially susceptible. Glyphosate was proposed as a possible factor in the increased risk to Clostridium botulinum infection in cattle in Germany over the past ten to fifteen years (Krüger *et al.*, 2013b). Pigs fed GMO corn and soy developed widespread intestinal inflammation that may have been due in part to glyphosate exposure (Carman *et al.*, 2013).

Celiac disease is associated with reduced levels of Enterococcus, Bifidobacteria and Lactobacillus in the gut and an overgrowth of pathogenic gram negative bacteria (Sanz *et al.*, 2011; Di Cagno *et al.*, 2011; Collado *et al.*, 2007). In (Di Cagno *et al.*, 2011), Lactobacillus, Enterococcus and Bifidobacteria were found to be significantly lower in fecal samples of children with celiac disease compared to controls, while levels of the pathogens, Bacteroides, Staphylococcus, Salmonella, a Shighella were elevated. In (Collado *et al.*, 2007), another study comparing the fecal material of celiac infants to healthy controls, Bacteroides, Clostridium and Staphylococcus were all found to be significantly higher (*p<*0.05). Sulfate-reducing bacterial counts were also elevated (*p<*0.05) (Nadal *et al.*, 2007; Collado *et al.*, 2007), an interesting observation which we will return to later in this paper. A significant reduction in Bifidobacteria was also found in (Nadal *et al.*, 2007). An increased excretion of the bacterial metabolites p-Cresol and phenol has also been recognized in association with celiac disease (Tamm, 1984). p-Cresol is produced via anaerobic metabolism of tyrosine by pathogenic bacteria such as Clostridium difficile (D'Ari and Barker, 1985). It is a highly toxic carcinogen, which also causes adverse effects on the central nervous system, the cardiovascular system, lungs, kidney and liver (Kelly *et al.*, 1994).

Probiotic treatments are recommended to aid in digestive healing in celiac disease. The proteolytic activity of Lactobacilli aids the breakdown of wheat into less allergenic forms. Ongoing research aims to produce gluten-containing sourdough breads fermented by Lactobacilli that can then serve as probiotics to help ameliorate the symptoms of celiac disease and allow celiac patients to consume wheat (Gobbetti *et al.*, 2007). Probiotic Lactobacilli produce the enzyme phytase which breaks down phytates that would otherwise deplete important minerals and other cations through chelation (Famularo *et al.*, 2005). Their activities would therefore improve absorption of these micronutrients, a known problem in celiac patients (Cavallaro *et al.*, 2004). Glyphosate itself also chelates rare minerals, a subject we will address in the section on nutritional deficiencies.

Probiotic treatment with Bifidobacteria has been shown to alleviate symptoms associated with celiac disease (Smecuol *et al.*, 2013; Whorwell *et al.*, 2006). Bifidobacteria suppress the pro-inflammatory milieu triggered by the microbiota of celiac patients (Medina *et al.*, 2008). Live cultures of Bifidobacterium lactis would promote healing of the gut if offered as treatment in conjunction with the gluten-free diet, or might even allow the celiac patient to consume modest amounts of gluten without damaging effects (Lindfors *et al.*, 2008). In this in vitro study, it was demonstrated that B. lactis reduced epithelial permeability and improved the integrity of the tight junctions in human colon cells.

In summary, celiac disease is associated with a reduced presence in the gut of commensal bacteria such as Lactobacilli and Bifidobacteria, which are known to be preferentially killed by glyphosate, and with an overabundance of C. difficile, which is known to be promoted by glyphosate exposure. Bifidobacteria and Lactobacilli are both capable of modifying gluten in such a way as to make it less allergenic, a feature that is being exploited in recent efforts to develop gluten-containing foods that may be safe for consumption by celiac patients. Probiotics containing live forms of these bacteria are also being actively marketed today.

## 3 CYP Enzyme impairment and sulfate depletion

As mentioned previously, glyphosate has been shown to suppress CYP enzymes in plants (Lamb *et al.*, 1998) and animals (Hietanen *et al.*, 1983). A study on rats demonstrated that glyphosate decreased the levels of CYP enzymes and monooxygenase activities in the liver and the intestinal activity of aryl hydrocarbon hydroxylase (Hietanen *et al.*, 1983).

CYP enzymes are essential for detoxification of many compounds in the liver (Lindros, 1997). Intraperitoneal exposure of rats to Roundup in acute doses over a short time interval induced irreversible damage to hepatocytes and elevated urinary markers of kidney disease. This was associated with lipid peroxidation and elevated levels of the inflammatory cytokine tumor necrosis factor (TNF-α) (El-Shenawy, 2009). CYP3A is constitutively expressed in human intestinal villi and plays an important role in drug metabolism (Cupp & Tracy, 1998). Celiac disease is associated with a decrease in the intestinal CYP3A (Lang *et al.*, 1996). This defect is restored by a gluten free diet.

Impaired gallbladder bile acid production (Colombato *et al.*, 1977) and biliary cirrhosis, an inflammatory liver disease characterized by obstruction of the bile duct (Dickey *et al.*, 1997), have been shown to co-occur with celiac disease. CYP enzymes are crucial in the production of bile acids (Lorbek *et al.*, 2012). An obligatory CYP enzyme in bile acid synthesis, CYP27A, has been identified as being identical to the mitochondrial vitamin D3 activating enzyme (Wikvall, 2001). In (Kemppainen *et al.*, 1999), 64% of men and 71% of women with celiac disease were found to be vitamin D3 deficient, manifested as low spinal bone mineral density. Celiac disease is associated with impaired gall bladder function and decreased pancreatic secretions (Brown *et al.*, 1987; Benini *et al.*, 2012) along with recurrent pancreatitis (Patel *et al.*, 1999). Abnormalities in bile acid secretion have been found in children suffering from celiac disease (Ejderhamn *et al.*, 1992). Celiac patients exhibit abnormally low synthesis of cholecystokinin (Deprez *et al.*, 2002), but it has also become apparent that the gall bladder is less responsive to stimulation of contraction by cholecystokinin (Brown *et al.*, 1987). A reversible defect of gallbladder emptying and cholecystokinin release has been identified in association with celiac disease (Maton *et al.*, 1985). These pathologies may be related to impaired CYP enzyme activity induced by glyphosate.

While it is clear that CYP enzymes play an important role in bile acid synthesis and in cholesterol homeostasis, the details have not yet been worked out (Lorbek *et al.*, 2012). However, some mouse knockout experiments produce embryonically lethal effects, pointing to the importance of these enzymes to biological systems. Disruption of Cyp7A1, involved in bile acid synthesis in mice, induces elevated serum cholesterol and early death.

A link has been established between celiac disease and non-alcoholic fatty liver, which is likely due to the liver's inability to export cholesterol sulfate through the bile acids due to impaired CYP enzymes (Lorbek *et al.*, 2012). This requires a private store of fats to house the excess cholesterol that cannot be exported in bile. This would also likely lead to insufficient sulfate supplies to the small intestine, and could result in impaired heparan sulfate synthesis in the glycosaminoglycans and subsequent pathologies. Heparan sulfate populating the glycosaminoglycans (GAGs) surrounding enterocytes is essential for the proper functioning of the small intestines. Leakage of both albumin and water in both the vasculature and tissues results when the negative charge is reduced due to insufficient sulfation of the polysaccharide units (Sunergren *et al.*, 1987). Vascular leakage may be a consequence of degradation of sulfated GAGs due to inflammatory agents (Klein *et al.*, 1992). A similar problem may occur in the kidneys leading to albumin loss into urine during nephrosis (Vernier *et al.*, 1983). Intestinal protein loss in inflammatory enteropathy associated with celiac disease may also be due to a deficiency in the sulfated GAGs (Murch *et al.*, 1993; Murch, 1995). A case study of three infants with congenital absence of enterocyte heparan sulfate demonstrated profound enteric protein loss with secretory diarrhoea and absorption failure, even though their intestines were not inflamed (Murch *et al.*, 1996).

In (Samsel and Seneff, 2013), a hypothesis was developed that glyphosate disrupts the transport of sulfate from the gut to the liver and pancreas, due to its competition as a similarly kosmotropic solute that also increases blood viscosity. (Kosmotropes are ions that induce “structure ordering” and “salting out” of suspended particles in colloids). Insufficient sulfate supply to the liver is a simple explanation for reduced bile acid production. The problem is compounded by impaired CYP enzymatic action and impaired cycling of bile acids through defective enterocytes in the upper small intestine. The catastrophic effect of loss of bile acids to the feces due to impaired reuptake compels the liver to adopt a conservative approach of significantly reduced bile acid synthesis, which, in turn, leads to gall bladder disease.

The protein, Nuclear factor κ-lightchain-enhancer of activated B cells (NF-κB) controls DNA transcription of hundreds of genes and is a key regulator of the immune response to infection (Tieri *et al.*, 2012). Light chains are polypeptide subunits of immunoglobulins. NF-κB responds to stimulation from bacterial and viral antigens, inflammatory cytokines like TNF-α, free radicals, oxidized LDL, DNA damage and UV light. The incidence of acute pancreatitis has been increasing in recent years (Bhatia, 2012), and it often follows billiary disease. A local inflammatory reaction at the site of injury coincides with an increase in the synthesis of hydrogen sulfide (H_2_S) gas. H_2_S regulates the inflammatory response by exciting the extracellular signal regulated (ERK) pathway, leading to production of NF-κB (Bhatia, 2012). We hypothesize that H_2_S, while toxic, is a source of both energy and sulfate for the pancreas, derived from sulfur-containing amino acids such as cysteine and homocysteine. Dehydroepiandrosterone (DHEA) sulfate, but not DHEA, inhibits NF-κB synthesis, suggesting that sulfate deficiency is a driver of inflammation (Iwasaki *et al.*, 2004).

While H_2_S is well known as a toxic gas through its inhibition of aerobic respiration, a recent paradigm shift in the research surrounding H_2_S has been inspired by the realization that it is an important signaling gas in the vasculature, on par with nitric oxide (Li *et al.*, 2011). H_2_S can serve as an inorganic source of energy to mammalian cells (Módis *et al.*, 2013). 3-mercaptopyruvate sulfurtransferae (3MST) is expressed in the vascular endothelium, and it produces H_2_S from mercaptopyruvate, an intermediary in the breakdown of cysteine (Kimura, 2011). Endogenously produced H_2_S derived from 3-mercaptopyruvate stimulates additional mitochondrial H_2_S production, which then is oxidized to thiosulfate via at least three different pathways (Ingenbleek and Kimura, 2013; Hildebrandt and Grieshaber, 2008; Goubern *et al.*, 2007), producing ATP. The inflammatory agent superoxide can act as substrate for the oxidation of H_2_S to sulfite and subsequently sulfate and the activated form, PAPS (Seneff *et al.*, 2012), but will likely induce oxidative damage in the pancreas, particularly, as we will see in section 7, if molybdenum deficiency impairs sulfite-to-sulfate synthesis.

Pancreatic beta cells express extraordinarily high levels of heparan sulfate, which is essential for their survival (Ziolkowski *et al.*, 2012), since it protects them from ROS-induced cell death. Because sulfate transport via the hepatic portal vein is likely disrupted by glyphosate, H_2_S, whether derived from sulfur-containing amino acids or supplied via diffusion following its production by sulfur-reducing bacteria in the gut, can become an important source of sulfur for subsequent sulfate production locally in the pancreatic cells. Pancreatic elastase is a serine protease that is needed to assist in protein degradation, but an overabundance can lead to autolysis of tissues (Ito *et al.*, 1998). Cholesterol sulfate inhibits pancreatic elastase (Ito *et al.*, 1998), so a deficiency in cholesterol sulfate supply due to impaired sulfate supply to the liver and impaired CYP function should increase the risk of tissue digestion by pancreatic enzymes, contributing to the loss of villi in the upper small intestine observed in celiac disease.

In the early 1990's a newly recognized disease began to appear, characterized by eosinophil infiltration into the esophagus, which manifested as dysphagia in adults and refractory reflux symptoms in children (Lucendo & Sánchez-Cazalilla, 2012). This disease, termed eosinophilic esophagitis (EOE), is associated with a Th2 immune profile and synthesis of the cytokine IL-13, which has direct cytotoxic effects on epithelial cells. A dose-dependent induction of eosinophilia by intratracheal delivery of IL-13 confirms its association with EOE (Mishra and Rothenberg, 2003). An association has been found between EOE and celiac disease (Leslie *et al.*, 2010). Patients with refractory celiac disease that is not corrected by dietary gluten restriction show an increased production of IL-13 in the gut (Gross *et al.*, 2013). The incidence of EOE has increased at alarming rates in Western countries in the last three decades (Furuta *et al.*, 2007; Liacouras *et al.*, 2011; Prasad *et al.*, 2009).

Glyphosate is highly corrosive to the esophageal epidermal lining, with upper GI tract injury observed in 94% of patients following glyphosate ingestion (Chang *et al.*, 1999). In (Zouaoui *et al.*, 2013), the most common symptoms in an acute response from glyphosate poisoning were oropharyngeal ulceration, nausea and vomiting. We hypothesize that glyphosate induces EOE via a systemic response as well as through direct contact. The pathogenesis of EOE is related to food sensitivities, but airborne exposure to chemicals in the lungs can also induce it, so it does not require physical contact to the allergen (Blanchard & Rothenberg, 2008). It is conceivable that glyphosate is responsible for the emergence of EOE.

The cytochrome P450 reductase (CPR) and cytochrome P450 (CP) enzyme system is essential for inducing nitric oxide release from organic nitrates (Li, 2006). The nitrate moiety is reduced while simultaneously oxidizing NADPH to NADP+. This system is invoked in organic nitrate drug treatment for cardiovascular therapy. The reaction depends on anaerobic, acidic conditions, a feature of venous rather than arterial blood. Since L-arginine is substrate for NO synthesis by endothelial nitric oxide synthase (eNOS) under oxidative conditions (Förstermann and Münze, 2006), it is likely that CPR and CP play an important role mainly in stimulating *venous* smooth muscle relaxation. Impaired venous relaxation would likely contribute to venous thrombosis, which is a well-established complication of celiac disease (Zenjari *et al.*, 1995; Marteau *et al.*, 1994, Grigg, 1999, Halfdanarson *et al.*, 2007).

In summary, celiac disease is associated with multiple pathologies in the digestive system, including impaired gall bladder function, fatty liver, pancreatitis, and EOE. We have argued here that many of these problems can be traced to impaired CYP function in the liver due to glyphosate exposure, leading to insufficient flow of bile acids through the circular pathway between the liver and the gut. This results in a system-wide depletion in sulfate, which induces inflammation in multiple organs to produce sulfate locally. A potential sulfur source for sulfate synthesis could be hydrogen sulfide gas, provided in part by the local breakdown of sulfur-containing amino acids like cysteine and homocysteine and in part by diffusion of the gas produced from inorganic dietary sources by sulfur-reducing bacteria in the large intestine. Impaired CYP enzyme function may also contribute to venous thrombosis, for which celiac disease is an established risk factor.

## 4 Retinoic acid, celiac disease and reproductive issues

In this section, we first establish that excess retinoic acid (RA) is a risk factor for celiac disease. We then show that excess RA leads to complications in pregnancy and teratogenic effects in offspring. Glyphosate has been shown to exhibit teratogenic effects in line with known consequences of excess RA exposure to the embryo, and we propose that the mechanism for this effect may be glyphosate's known disruption of CYP enzymes (Samsel & Seneff, 2013), which are involved in RA catabolism. This then links glyphosate to increased risk to celiac disease via its direct effects on RA. And it identifies a possibly important factor in the association of celiac disease with reproductive issues. We also discuss other adverse effects of excess retinoic acid and a possible relationship to impaired sulfate supply to the gut.

In celiac disease, T cells develop antibody responses against dietary gluten, a protein present in wheat (Jabri & Sollid, 2009). RA, a metabolite of vitamin A, has been shown to play a critical role in the induction of intestinal regulatory responses (Mora *et al.*, 2008; Coombes *et al.*, 2007; Mucida *et al.*, 2007). The peptide in gluten, A-gliadin p31-43, induces interleukin 15 (IL-15), a key cytokine promoting T-cell activation (Hershko & Patz, 2008). RA synergizes with high levels of IL-15 to promote JNK phosphorylation (Nanda, 2011; DePaolo *et al.*, 2011), which potentiates cellular apoptosis (Putcha *et al.*, 2003). IL-15 is a causative factor driving the differentiation of precursor cells into anti-gluten CD4+ and CD8+ Th1 cells in the intestinal mucosa. Furthermore, in (DePaolo *et al.*, 2011), it was discovered that RA exhibits an unanticipated co-adjuvant property to induce Th1 immunity to antigens during infection of the intestinal mucosa with pathogens. Retinoic acid has also been shown to directly suppress transglutaminase activity, another way in which it would negatively impact celiac disease (Thacher *et al.*, 1985). Thus, it is becoming clear that excess exposure to RA would increase risk to celiac disease, and warnings have been issued regarding potential adverse effects of RA supplements on celiac disease.

It is well established that high RA levels leads to teratogenic effects both in human and experimental models. Brain abnormalities such as microcephaly, impairment of hindbrain development, mandibular and midfacial underdevelopment, and cleft palate are all implicated (Sulik *et al.*, 1988; Clotman *et al.*, 1998). Women with celiac disease are known to have higher rates of infertility, miscarriages, and birth defects in their offspring (Freeman, 2010; Martinelli *et al.*, 2000; Dickey *et al.*, 1996; Collin *et al.*, 1996). Excess RA could be a significant factor in these complications.

A possible mechanism by which glyphosate might induce excess RA is via its interference with the CYP enzymes that metabolize RA. There are at least three known CYPs (CYP26A1, CYP26B1 and CYP26C1) that catabolize RA, and they are active in both the embryo and the adult (Taimi *et al.*, 2004). A 1/5000 dilution of glyphosate was sufficient to induce reproducible malformations characteristic of RA exposure in frog embryos (Paganelli *et al.*, 2010). Pathologies included shortening of the trunk, reduction in the size of the head, abnormally small eyes or the presence of only one eye (cyclopia), and other craniofacial malformations in the tadpole. Glyphosate's toxicity to tadpoles has been well demonstrated, as it killed nearly 100% of larval amphibians exposed in experimental outdoor pond mesocosms (Relyea, 2005).

According to official records, there has been a recent 4-fold increase in developmental malformations in the province of Chaco, Argentina, where glyphosate is used massively on GMO monocrops of soybeans (Carrasco, 2013). In Paraguay, 52 cases of malformations were reported in the offspring of women exposed during pregnancy to agrochemicals, including anencephaly, microcephaly, facial defects, cleft palate, ear malformations, polydactily, and syndactily (Benítez-Leite *et al.*, 2009). In in vitro studies on human cell lines, DNA strand breaks, plasma membrane damage and apoptosis were observed following exposure to glyphosate-based herbicides (Gasnier *et al.*, 2009). Another factor in teratogenetic effects of glyphosate may be the suppression of the activity of androgen-to-estrogen conversion by aromatase, a CYP enzyme (Gasnier *et al.*, 2009).

Ingested vitamin A, a fat-soluble vitamin, is delivered to the blood via the lymph system in chylomicrons, and excess vitamin A is taken up by the liver as retinoic acid for catabolism by CYP enzymes (Russell, 2000). Any remaining retinoic acid that is not catabolized is exported inside LDL particles, and it lingers much longer as retinyl esters in the vasculature in this form (Krasinski *et al.*, 1990). Excess retinoic acid is more readily stored in this way in LDL particles in the elderly. Vitamin A toxicity can lead to fatty liver and liver fibrosis (Russell, 2000) as well as hypertriglyceridemia (Ellis *et al.*, 1986). Vitamin A has a negative effect on cholesterol sulfate synthesis (Jetten *et al.*, 1989), which might negatively impact the liver's ability to maintain adequate supplies of cholesterol sulfate for the bile acids, and therefore also interfere with the supply of cholesterol sulfate to the gastrointestinal tract.

In summary, glyphosate's disruption of the CYP enzymes responsible for RA catabolism could lead to an excess bioavailability of RA that could contribute adversely to celiac disease, as well as damaging the liver and leading to teratogenic effects in offspring of exposed individuals.

In addition to higher risk to birth defects, individuals with celiac disease have increased risk to infertility (Meloni *et al.*, 1999; Farthing *et al.*, 1982). Increased incidence of hypogonadism, infertility and impotence was observed in a study of 28 males with celiac disease (Farthing *et al.*, 1982). Marked abnormalities of sperm morphology and motility were noted, and endocrine dysfunction was suggested as a probable cause. In studies conducted on Sertoli cells in prepubertal rat testis, exposure to Roundup induced oxidative stress leading to cell death (de Liz Oliveira Cavalli *et al.*, 2013). Roundup induced the opening of L-type voltage dependent calcium channels as well as ryanodine receptors, initiating ER stress and leading to calcium overload and subsequent necrosis. Glutathione was depleted due to upregulation of several glutathione-metabolizing enzymes. This suggests that Roundup would interfere with spermatogenesis, which would impair male fertility.

## 5 Cobalamin deficiency

Untreated celiac disease patients often have elevated levels of homocysteine, associated with folate and/or cobalamin deficiency (Saibeni *et al.*, 2005; Dickey *et al.*, 2008). Species of Lactobacillus and Bifidobacterium have the capability to biosynthesize folate (Rossi *et al.*, 2011), so their disruption by glyphosate could contribute to folate deficiency. Malabsorption in the proximal small intestine could also lead to iron and folate deficiencies. Cobalamin was originally thought to be relatively spared in celiac disease because its absorption is mostly through the terminal ileum, which is unaffected by celiac disease. However, a recent study found that cobalamin deficiency is prevalent in celiac patients. 41% of the patients studied were found to be deficient in cobalamin (<220 ng/L), and 31% of these cobalamin-deficient patients also had folate deficiency (Dahele & Ghosh, 2001). Either cobalamin or folate deficiency leads directly to impaired methionine synthesis from homocysteine, because these two vitamins are both required for the reaction to take place. This induces hyperhomocysteinemia (Refsum *et al.*, 2001), an established risk factor in association with celiac disease (Hadithi *et al.*, 2009). Long-term cobalamin deficiency also leads to neurodegenerative diseases (Herrmann & Obeid, 2012).

Because a deficiency in cobalamin can generate a large pool of methyl-tetrahydrofolate that is unable to undergo reactions, cobalamin deficiency will often mimic folate deficiency. Cobalamin requires cobalt, centered within its corrin ring, to function. We depend upon our gut bacteria to produce cobalamin, and impaired cobalt supply would obviously lead to reduced synthesis of this critical molecule. Glyphosate is known to chelate +2 cations such as cobalt. Glyphosate complexes with cobalt as a dimer [Co(glyphosate)2]3 in fifteen different stereoisomeric configurations, and it is facile at switching among the different stereoisomers, an unusual kinetic property compared to most Co(III) systems (Cusiel, 2005).

In fact, studies have revealed that glyphosate inhibits other cytosolic enzymes besides EPSP synthase in plants and microbes that also activate steps in the shikimate pathway (Ganson and Jensen, 1988; Bode *et al.*, 1984). Glyphosate potently inhibits three enzymes in the shikimate pathway in yeast (Bode *et al.*, 1984). It has been confirmed that these other enzymes depend upon cobalt as a catalyst, and glyphosate inhibition works through competitive cobalt binding and interference with cobalt supply (Ganson and Jensen, 1988). It has also been proposed that chelation by glyphosate of both cobalt and magnesium contributes to impaired synthesis of aromatic amino acids in Escherichia coli bacteria (Hoagland and Duke, 1982). Thus, it is plausible that glyphosate similarly impairs cobalamin function in humans by chelating cobalt.

## 6 Anemia and iron

Anemia is one of the most common manifestations of celiac disease outside of the intestinal malabsorption issues (Halfdanarson *et al.*, 2007; Bottaro *et al.*, 1999), and is present in up to half of diagnosed celiac patients. Celiac patients often have both cobalamin and folate deficiency, which can cause anemia, but iron deficiency may be the most important factor (Hershko & Patz, 2008). Celiac patients often don't respond well to iron treatment.

Glyphosate's chelating action can have profound effects on iron in plants (Eker *et al.*, 2006; Bellaloui *et al.*, 2009). Glyphosate interferes with iron assimilation in both glyphosate-resistant and glyphosate-sensitive soybean crops (Bellaloui *et al.*, 2009). It is therefore conceivable that glyphosate's chelation of iron is responsible for the refractory iron deficiency present in celiac disease.

Erythropoietin (EPO), also called hematopoietin, is a cytokine produced by interstitial fibroblasts in the kidney that regulates red blood cell production. Low EPO levels, leading to a low turnover rate of red blood cells, is a feature of celiac disease (Bergamaschi *et al.*, 2008; Hershko & Patz, 2008). This can lead to megaloblastic anemia, where red blood cells are large (macrocytic) and reduced in number due to impaired DNA synthesis. A recent hematological study on mice exposed to Roundup at subacute levels for just 15 days revealed an anemic syndrome in both male and female mice, with a significant reduction in the number of erythrocytes and in hemoglobin, reduced hematocrit and increased mean corpuscular volume, indicative of macrocytic anemia (Jasper *et al.*, 2012).

## 7 Molybdenum deficiency

Molybdenum deficiency is rarely considered in diagnoses, as it is only needed in trace amounts. However, molybdenum is essential for at least two very important enzymes: sulfite oxidase and xanthine oxidase. Sulfite oxidase converts sulfite, a highly reactive anion, to sulfate, which is much more stable. Sulfite is often present in foods such as wine and dried fruits as a preservative. Sulfate plays an essential role in the sulfated proteoglycans that populate the extracellular matrices of nearly all cell types (Turnbull *et al.*, 2001; Murch *et al.*, 1993; Murch, 1995). So, impaired sulfite oxidase activity leads to both oxidative damage and impaired sulfate supplies to the tissues, such as the enterocytes in the small intestine. The excess presence of sulfur-reducing bacteria such as Desulfovibrio in the gut in association with celiac disease (Collado *et al.*, 2007; Nadal *et al.*, 2007) could be protective, because these bacteria can reduce dietary sulfite to hydrogen sulfide, a highly diffusable gas that can migrate through tissues to provide a source of sulfur for sulfate regeneration at a distant site, as previously discussed. These distal sites could reoxidize the H_2_S through an alternative pathway that does not require molybdenum for sulfur oxidation (Ingenbleek and Kimura, 2013).

Xanthine oxidase (XO) produces uric acid from xanthine and hypoxanthine, which are derived from purines. It is activated by iron, which, as we have seen, is often intractably deficient in association with celiac disease. Impaired XO activity would be expected to drive purines towards other degradation pathways. Adenosine deaminase (ADA), a cytoplasmic enzyme that is involved in the catabolism of purine bases, is elevated in celiac disease, and is therefore a useful diagnostic marker (Cakal *et al.*, 2010). In fact, elevation of ADA is correlated with an increase in several inflammatory conditions. Impaired purine synthesis is expected in the context of cobalamin deficiency as well, because methyl melonlyl CoA mutase depends on catalytic action by cobalamin (Allen *et al.*, 1993). Decreased purine synthesis results in impaired DNA synthesis, which then leads to megaloblastic anemia (Boss, 1985), due to slowed renewal of RBC's from multipotent progenitors, a problem that is compounded by suppressed EPO activity (Bergamaschi *et al.*, 2008), a feature of celiac disease.

A remarkable recent case of a three-month old infant suffering from molybdenum deficiency links several aspects of glyphosate toxicity together, although glyphosate exposure was not considered as a possible cause in this case (Boles *et al.*, 1993). This child presented with microcephaly, developmental delay, severe irritability, and lactic acidosis. Lactic acidosis is a striking feature of intentional glyphosate poisoning induced by drinking Roundup (Zouaoui *et al.*, 2013; Beswick & Millo, 2011), and it suggests impaired oxidative respiration, as is seen in E. coli exposed to glyphosate (Lu *et al.*, 2013). In vitro studies of glyphosate in the formulation Roundup have demonstrated an ability to disrupt oxidative respiration by inducing mitochondrial swelling and inhibiting mitochondrial complexes II and III (Peixoto, 2005). This would explain a massive build-up of lactic acid following ingestion of Roundup, due to a switch to anaerobic metabolism. Glyphosate has also been shown to uncouple mitochondrial phosphorylation in plants (Haderly *et al.*, 1977; Ali & Fletcher, 1977).

As has been stated previously, microcephaly is a feature of excess RA, which could be induced by glyphosate due to its inhibitory action on CYP enzymes. In the case study on molybdenum deficiency (Boles *et al.*, 1993), urinary sulfite levels were high, indicative of defective sulfite oxidase activity. Serum hypouricemia was also present, indicative of impaired XO activity. So, the induction of excess RA, depletion of molybdenum, and lactic acidosis by glyphosate provide a plausible environmental factor in this case.

One final aspect of molybdenum deficiency involves nitrate metabolism. As a source of nitric oxide, inorganic nitrite regulates tissue responses to ischemia. While nitrate reductase activity has been known to be a capability of microbes for many years, it has only recently been realized that mammals also possess a functioning nitrate reductase capability, utilizing a molybdenum-dependent enzyme to produce nitrite from nitrate (Jansson *et al.*, 2008). Molybdenum deficiency would impair this capability, likely contributing to the higher risk to venous thrombosis observed in celiac disease (Zenjari *et al.*, 1995; Marteau *et al.*, 1994, Grigg, 1999). This could also explain the excess nitrates in the urine observed in association with celiac disease (Högberg *et al.*, 2011).

## 8 Selenium and thyroid disorders

Autoimmune thyroid disease is associated with celiac disease (Collin *et al.*, 2002; Valentino *et al.*, 2002). In (Valentino *et al.*, 2002), up to 43% of patients with Hashimoto's thyroiditis showed signs of mucosal T-cell activation typical of celiac disease. Selenium, whose deficiency is associated with celiac disease (Hinks *et al.*, 1984), plays a significant role in thyroid hormone synthesis, secretion and metabolism, and selenium deficiency is therefore a significant factor in thyroid diseases (Sher, 2000; Chanoine *et al.*, 2001; Khrle, 2013).

Selenium is required for the biosynthesis of the “twenty first amino acid,” selenocysteine. Twenty five specific selenoproteins are derived from this amino acid. Selenium deficiency can lead to an impairment in immune function and spermatogenesis in addition to thyroid function (Papp *et al.,* 2007). One very important selenoprotein is glutathione peroxidase, which protects cell membranes and cellular components against oxidative damage by both hydrogen peroxide and peroxynitrite (ONOO^–^) (Prabhakar *et al.*, 2006).

Wheat can be a good source of selenoproteins. However, the content of selenium in wheat can range from sufficient to very low, depending upon soil physical conditions. Soil compaction, which results from modern practices of “no till” agriculture (Huggins & Reganold, 2008), can lead to both reduced selenium content and a significant increase in arsenic content in the wheat (Zhao *et al.*, 2007). Since glyphosate has been shown to deplete sulfur in plants (Saes Zobiole *et al.*, 2010), and selenium is in the same column of the periodic table as sulfur, it is likely that glyphosate also disrupts selenium uptake in plants. A gluten-free diet will guarantee, however, that no selenium is available from wheat, inducing further depletion of selenoproteins, and therefore increasing the risk to immune system, thyroid and infertility problems in treated celiac patients.

The gut bacterium Lactobacillus, which is negatively impacted by glyphosate (Shehata *et al.*, 2013) and depleted in association with celiac disease (Di Cagno *et al.*, 2011), is able to fix inorganic selenium into more bioavailable organic forms like selenocysteine and selenomethionine (Pessione, 2012). Selenocysteine is present in the catalytic center of enzymes that protect the thyroid from free radical damage (Triggiani *et al.*, 2009). Free radical damage would lead to apoptosis and an autoimmune response (Tsatsoulis, 2002). Glyphosate's disruption of these bacteria would lead to a depletion in the supply of selenomethionine and selenocysteine. Methionine depletion by glyphosate (Nafziger *et al.*, 1984) would further compound this problem.

**Figure 3:**
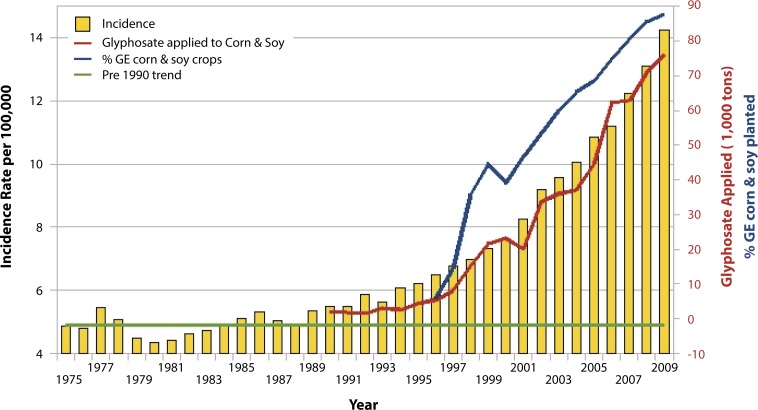
Thyroid cancer incidence rate plotted against glyphosate applied to U.S. corn & soy crops (R=0.988, *p≤*7.612e-09) along with % GE corn & soy crops (R=0.9377, *p≤*2.152e-05). Sources: USDA:NASS; SEER. (Figure courtesy of Nancy Swanson).

Thus, there are a variety of ways in which glyphosate would be expected to interfere with the supply of selenoproteins to the body, including its effects on Lactobacillus, its depletion of methionine, the no-till farming methods that are possible because weeds are killed chemically, and the likely interference with plant uptake of inorganic selenium. This aligns well with the observed higher risk of thyroid problems in association with celiac disease, in addition to infertility problems and immune issues, which are discussed elsewhere in this paper. Further support for an association between glyphosate and thyroid disease comes from plots over time of the usage of glyphosate in the U.S. on corn and soy time-aligned with plots of the incidence rate of thyroid cancer in the U.S., as shown in Figure 3.

## 9 Indole and kidney disease

The prevalence of kidney disease and resulting dialysis is increasing worldwide, and kidney disease is often associated with increased levels of celiac disease autoantibodies. Kidney disease and thyroid dysfunction are intimately connected (Iglesias & Díez, 2009). A population-based study in Sweden involving nearly 30,000 people with diagnosed celiac disease determined that there was nearly a three-fold increased risk for kidney failure in this population group (Welander *et al.*, 2012).

Inflammation plays a crucial role in kidney disease progression (Tonelli *et al.*, 2005; Bash *et al.*, 2009; Rodriguez-Iturbe *et al.*, 2010). Chronic kidney disease develops as a consequence of assaults on the kidney from inflammatory agents, brought on by the induction of pro-inflammatory cytokines and chemokines in the kidney. The toxic phenol p-Cresol sulfate, as well as indoxyl sulfate, a molecule that is chemically similar to p-Cresol, have been shown to induce activation of many of these cytokines and chemokines (Sun *et al.*, 2012). p-Cresol and indoxyl sulfate both decrease endothelial proliferation and interfere with wound repair (Dou *et al.*, 2004). p-Cresol is produced by the pathogenic bacterium C. difficile, and indoxyl sulfate, derived from indole through sulfation in the liver (Banoglu & King, 2002), accumulates at high levels in association with chronic kidney disease (Niwa, 2010).

The aromatic amino acid tryptophan contains an indole ring, and therefore disruption of tryptophan synthesis might be expected to generate indole as a by-product. Indeed, glyphosate has been shown to induce a significant increase in the production of indole-3-acetic acid in yellow nutsedge plants (Caal *et al.*, 1987). Indole is produced by coliform microorganisms such as E. coli under anaerobic conditions. Glyphosate induces a switch in E. coli from aerobic to anaerobic metabolism due to impaired mitochondrial ATP synthesis (Lu *et al.*, 2013; Samsel & Seneff, 2013), which would likely result in excess production of indole. Besides, E. coli, many other pathogenic bacteria can produce indole, including Bacillus, Shigella, Enterococcus, and V. cholerae (Lee & Lee, 2010). At least 85 different species of both Gram-positive and Gram-negative bacteria produce indole, and its breakdown by certain bacterial species depends on CYP enyzmes (Lee & Lee, 2010). Feeding indole to rats deprived of sulfur metabolites leads to macrocytic anemia (Roe, 1971). Indole is an important biological signaling molecule among microbes (Lee & Lee, 2010). Indole acetic acid inhibits the growth of cobalamin-dependent microorganisms, which then causes macrocytic (pernicious) anemia in the host due to cobalamin deficiency (Drexler, 1958).

Experiments on exposure of mouse fetuses to indole-3-acetic acid have shown that it dramatically induces microcephaly in developing fetuses exposed at critical times in development (Furukawa *et al.*, 2007). A case study found celiac disease associated with microcephaly and developmental delay in a 15-month-old girl (Bostwick *et al.*, 2001; Lapunzina, 2002). A gluten-free diet restored head growth. The authors suggested that poor head growth might precede other manifestations of celiac disease in infants. A study on plants demonstrated a concentration gradient of indole-3-acetic acid in the plant embryo, similar to the gradient in retinoic acid that controls fetal development in mammals (Uggla *et al.*, 1996). This alternative may be another way in which glyphosate would promote microcephaly.

Thus, solely through its effect on indole production and indole catabolism in gut bacteria, chronic glyphosate exposure would be expected to lead to cobalamin deficiency, pernicious anemia, microcephaly in a fetus during pregnancy, and kidney failure. p-Cresol supply by overgrown pathogens like C. difficile would likely contribute in a similar way as indole, due to its similar biochemical and biophysical properties.

## 10 Nutritional deficiencies

The damaged villi associated with celiac disease are impaired in their ability to absorb a number of important nutrients, including vitamins B6, B12 (cobalamin) and folate, as well as iron, calcium and vitamins D and K (Hallert *et al.*, 2002). Thus, long-term celiac disease leads to major deficiencies in these micronutrients. Cobalamin deficiency has been well addressed previously. We have also already mentioned the chelation of trace minerals by phytates and by glyphosate. However, other factors may be at play as well, as discussed here.

Glyphosate disrupts the synthesis of tryptophan and tyrosine in plants and in gut bacteria, due to its interference with the shikimate pathway (Lu *et al.*, 2013; María *et al.*, 1996), which is its main source of toxicity to plants. Glyphosate also depletes methionine in plants and microbes. A study on serum tryptophan levels in children with celiac disease revealed that untreated children had significantly lower ratios of tryptophan to large neutral amino acids in the blood, and treated children also had lower levels, but the imbalance was less severe (Hernanz & Polanco, 1991). The authors suggested a metabolic disturbance in tryptophan synthesis rather than impaired absorption, as other similar amino acids were not deficient in the serum. It was proposed that this could lead to decreased synthesis of the monoamine neurotransmitter, serotonin, in the brain associated with behavior disorders in children with celiac disease, such as depression (Koyama & Melzter, 1986). Deficiencies in tyrosine and methionine were also noted (Hernanz & Polanco, 1991). “Functional dyspepsia” is an increasing and mainly intractable problem in the Western world, which is estimated to affect 15% of the U.S. population (Saad & Chey, 2006). Dyspepsia, a clinical symptom of celiac disease, is likely mediated by excess serotonin synthesis following ingested tryptophan-containing foods (Manocha *et al.*, 2012).

Serotonin (5-hydroxytryptamine or 5-HT) is produced by enterochromaffin (EC) cells in the gut and is an important signaling molecule for the enteric mucosa (Kim *et al.*, 2001). EC cells are the most numerous neuroendocrine cell type in the intestinal lumen, and they regulate gut secretion, motility, pain and nausea by activating primary afferent pathways in the nervous system (Chin *et al.*, 2012). Serotonin plays an important role in activating the immune response and inflammation in the gut, and also induces nausea and diarrhea when it is overexpressed. Anaerobic bacteria in the colon convert sugars into short-chain fatty acids, which can stimulate 5-HT release from EC cells (Fukumoto *et al.*, 2003; Grider & Piland, 2007). This is likely an important source of fats to the body in the case of a low-fat diet induced by impaired fatty acid metabolism due to insufficient bile acids.

The number of 5-HT expressing EC cells in the small intestine is increased in association with celiac disease, along with crypt hyperplasia (Wheeler & Challacombe, 1984; Challacombe *et al.*, 1977), and, as a consequence, serotonin uptake from dietary sources of tryptophan is greatly increased in celiac patients (Erspamer, 1986). Postprandial dyspepsia is associated in celiac disease with increased release of 5-HT, and this may account for the digestive symptoms experienced by celiac patients (Coleman *et al.*, 2006). An explanation for these observations is that a chronic tryptophan insufficiency due to the impaired ability of gut bacteria to produce tryptophan induces aggressive uptake whenever dietary tryptophan is available.

Glyphosate forms strong complexes with transition metals, through its carboxylic, phosphonic, and amino moieties, each of which can coordinate to metal ions, and it can also therefore form complexes involving two or three atoms of the targeted transition metal (Madsen *et al.*, 1978; Motekaitis & Martell, 1985; Undabeytia *et al.*, 2002). This means that it is a metal chelator par excellence. One can expect, therefore, deficiencies in multiple transition (trace) metals, such as iron, copper, cobalt, molybdenum, zinc and magnesium in the presence of glyphosate. Glyphosate has been shown to reduce levels of iron, magnesium, manganese and calcium in non-GMO soybean plants (Cakmak *et al.*, 2009). We have already discussed iron, selenium, cobalt and molybdenum deficiencies in association with celiac disease.

Zinc deficiency seems to be a factor in celiac disease, as a recent study of 30 children with celiac disease demonstrated a significantly reduced serum level of zinc (0.64 vs 0.94 µg/mL in controls) (Singhal *et al.*, 2008). Copper deficiency is a feature of celiac disease (Halfdanarson *et al.*, 2009), and copper is one of the transition metals that glyphosate binds to and chelates (Madsen, 1978; Undabeytia, 2002). Confirmed magnesium deficiency in celiac disease has been shown to be due to significant loss through the feces (Goldman *et al.*, 1962). This would be expected through binding to phytates and/or glyphosate. A study of 23 patients with gluten-sensitive enteropathy to assess magnesium status revealed that only one had *serum* magnesium levels below the normal range, whereas magnesium levels in *erythrocytes* and *lymphocytes* was markedly below normal, and this was associated with evidence of osteoporosis due to malabsorption (Rude and Olerich, 1996). Daily treatment with MgCl_2_ or Mg lactate led to a significant increase in bone mineral density, and was correlated with a rise in RBC Mg^2+^.

A recent study investigated the status of 25(OH) vitamin D3 in adults and children with celiac disease (Lerner *et al.*, 2012). It was determined that vitamin D3 deficiency was much more prevalent in the adults than in the children, suggesting a deterioration in vitamin D3 serum levels with age. This could be explained by a chronic accumulation of glyphosate, leading to increasingly impaired vitamin D3 activation in the liver. The liver converts 1,25(OH) vitamin D3 to the active form, 25(OH) vitamin D3, using CYP27A (Ponchon *et al.*, 1969; Sakaki *et al.*, 2005), which might be disrupted by glyphosate exposure, given its known interference with CYP function in mice (Hietanen *et al.*, 1983). On a broader level, this might also explain the recent epidemic in the U.S. in vitamin D3 deficiency (Holick, 2005).

Another issue to consider is whether the food being consumed by celiac patients is itself depleted in nutrients. This is likely the case for the transgenic Roundup-Ready crops that increasingly supply the processed food industry. A recent study on the effects of glyphosate on Roundup-Ready soy revealed a significant effect on growth, as well as an interference with the uptake of both macronutrients and micronutrients (Saes Zobiole *et al.*, 2010). Transgenic soybeans exposed to glyphosate are often affected by a “yellow flashing” or yellowing of the upper leaves, and an increased sensitivity to water stress. An inverse linear relationship was observed between glyphosate dosage and levels of the macronutrients, sodium, calcium, sulfur, phosphorus, potassium, magnesium, and nitrogen, as well as the micronutrients, iron, zinc, manganese, copper, cobalt, molybdenum, and boron. Glyphosate's ability to form insoluble metal complexes likely mediates these depletions (Glass, 1984). Glyphosate also interferes with photosynthesis, as reflected in several measures of photosynthesis rate (Saes *et al.*, 2010) and reductions in chlorophyll (Ali & Fletcher, 1977; Kitchen *et al.*, 1981). This could be due to depletion of zinc and manganese, since chloroplasts require these micronutrients to function well (Homann, 1967; Thompson & Weier, 1962).

## 11 Cancer

Chronic inflammation, such as occurs in celiac disease, is a major source of oxidative stress, and is estimated to account for 1/3 of all cancer cases worldwide (Ames *et al.*, 1993; Coussens & Werb, 2002). Oxidative stress leads to DNA damage and increased risk to genetic mutation. Several population-based studies have confirmed that patients with celiac disease suffer from increased mortality, mainly due to malignancy (Nielsen *et al.*, 1985; Logan *et al.*, 1989; Pricolo *et al.*, 1998; Cottone *et al.*, 1999; Corrao *et al.*, 2001; Green *et al.*, 2003). These include increased risk to non-Hodgkin's lymphoma, adenocarcinoma of the small intestine, and squamous cell carcinomas of the esophagus, mouth, and pharynx, as well as melanoma. The non-Hodgkin's lymphoma was not restricted to gastrointestinal sites, and the increased risk remained following a gluten-free diet (Green *et al.*, 2003).

Celiac disease is associated with a lifelong risk of any malignancy between 8.1 and 13.3%, with the risk for non-Hodgkin's lymphoma alone being 4.3 to 9.6% (Matheus-Vliezen *et al.*, 1994; Egan *et al.*, 1995). This risk is 19-fold higher than the risk in the general population. Selenium deficiency in association with celiac disease may be a significant factor in the increased cancer risk. Selenium deficiency is associated with increased risk to several cancers, and selenium supplements are beneficial in reducing the incidence of liver cancer and decreasing mortality in colorectal, lung and prostate cancer (Nelson *et al.*, 1999; Björnstedt *et al.*, 2010).

Children with celiac disease, whether or not they are on a gluten-free diet, exhibit elevated urinary biomarkers of DNA damage (Zaflarska-Popawska *et al.*, 2010). Human colon carcinoma cells exposed to peptides extracted from wheat responded with a sharp increase in the GSSG/GSH ratio (ratio of oxidized to reduced glutathione), a well-established indicator of oxidative stress (Rivabene, 1999). The authors did not provide information as to whether the wheat plants were exposed to glyphosate, but they did suggest that this effect could explain the increased risk to intestinal cancer associated with celiac. Intriguingly, studies on pea plants have shown that *glyphosate* induces a sharp increase in the GSSG/GSH ratio in plants (Miteva *et al.*, 2003), which suggests that glyphosate contamination could explain the results observed in (Rivabene, 1999).

Interestingly, it was noted in 1996 that the incidence of both non-Hodgkin's lymphoma and melanoma had been rising sharply worldwide in recent decades, and so it was decided to investigate whether there might be a link between the two cancers associated with sunlight exposure. Surprisingly, the authors found an *inverse* relationship between non-Hodgkin's lymphoma and UV exposure. More recently, such UV protection has been reaffirmed in a review of epidemiologic studies on the subject (Negri, 2010). This suggests that vitamin D3 is protective, so vitamin D3 deficiency due to impaired CYP function in the liver could be contributory to increased risk in celiac disease.

The incidence of non-Hodgkins lymphoma has increased rapidly in most Western countries over the last few decades. Statistics from the American Cancer Society show an 80% increase since the early 1970's, when glyphosate was first introduced on the market.

While there have been only a few studies of lymphoma and glyphosate, nearly all have indicated a potential relationship (Vigfusson & Vyse, 1980; Pavkov & Turnier, 1986; Hardell & Eriksson, 1999; McDuffie *et al.*, 2001; De Roos *et al.*, 2003). A dose-response relationship for non-Hodgkin's lymphoma was demonstrated in a cross-Canada study of occupational exposure to glyphosate in men (McDuffie *et al.*, 2001), and a larger study in the U.S. noted a similar result (De Roos *et al.*, 2003). A population-based study in Sweden showed an increased risk to non-Hodgkins lymphoma upon prior exposure to herbicides and fungicides but not insecticides (Hardell & Eriksson, 1999). Glyphosate exposure resulted in an odds ratio of 2.3, although the number of samples was small, and the authors suggested that further study is necessary. A study on mice showed increases in carcinoma, leukemia and lymphoma (Pavkov & Turnier, 1986) and an in vitro mutagenic test on human lymphocytes revealed increased sister-chromatid exchanges (Vigfusson & Vyse, 1980) upon exposure to glyphosate.

## 12 Proposed transglutaminase-glyphosate interactions

Establishing the mechanism by which glyphosate might promote autoantibodies to transglutaminase is a challenging task, not because this possibility seems unlikely but rather because multiple disruptions are plausible. In this section, we present evidence from the research literature that supports various hypotheses for the interaction of glyphosate with the transglutaminase enzymatic pathways. The definitive studies that clarify which of these hypotheses is correct have yet to be conducted.

Celiac disease is thought to be primarily caused by ingestion of wheat gluten proteins, particularly gliadin, due to a high concentration of proline- and glutamine-rich sequences, which imparts resistance to degradation by proteases. Transglutaminase autoimmunity arises when specific epitopes of wheat gliadin activate sensitized T-cells which then stimulate B-cell synthesis of IgA or IgM autoantibodies to transglutaminase. Transglutaminase bound to gliadin can induce false recognition by a T-cell.

Transglutaminase acts on gluten in wheat to form crosslinks between glutamine residues and lysine residues, producing ammonia as a by-product. Ammonia is known to induce greater sensitivity to glyphosate in plants, and it is common practice to apply ammonium sulfate simultaneously with glyphosate for this reason (Nalewaja & Matysiak, 1993). This enhanced effect is due to ammonium binding to glyphosate at three sites – one on the carbonyl group and two on the phosphonyl group, which displaces cations such as calcium and endows glyphosate with enhanced reactivity.

Transglutaminase sometimes only achieves half of its intended reaction product, by converting a glutamine residue to glutamate, and leaving lysine intact, thus not producing the desired crosslink. It has been established that gluten fragments containing “deamidated glutamine” residues instead of the crosslinks are much more highly allergenic than those that contain the crosslinks (Dørum *et al.*, 2010; Qiao *et al.*, 2005). These have been referred to as “celiac disease T-Cell epitopes.” T-cells of celiac patients preferentially recognize epitopes that are augmented with negatively charged deamidated glutamine residues – the product of the reaction when the lysine linkage does not occur. Thus, if there is a mechanism by which glyphosate interferes with crosslink formation, this would explain its ability to enhance gluten sensitivity.

A clue can be found from the research literature on glyphosate sensitivity in plants, where it has been determined that the substitution of a lysine residue in a critical locale in EPSP synthase greatly increases sensitivity to glyphosate (Selvapandiyan *et al.*, 1995). Lysine's NH3+ group is highly reactive with negatively charged ions, and this makes it a common constituent of DNA binding proteins due to its ability to bind to phosphates in the DNA backbone. Glyphosate contains a phosphonyl group that binds easily to ammonia and behaves as a phosphate mimetic. It also contains a carboxyl group that substitutes well for the carboxyl group of glutamate, the intended reaction partner.

Thus, it seems possible that glyphosate would be drawn to the ammonia released when the glutamine residue is deamidated by transglutaminase, and then the ammonium glyphosate would react with the lysine residue, releasing the ammonia and resulting in the binding of glyphosate to the lysine residue. This would yield a gluten fragment bound to glyphosate that is likely highly allergenic. An analogous EPSP synthase-EPSP-glyphosate ternary complex has been identified in numerous studies on the physiology of glyphosate in plants (Sammons *et al.*, 1995).

Research in the food industry has concerned producing breads that, while not gluten free, may contain forms of gluten to which celiac patients are less sensitive. Such research has revealed that enzymatic modification to promote methionine binding to glutamine reduces IgA immunoreactivity (Cabrera-Chávez *et al.*, 2010). Whether methionine binding to glutamine residues in wheat takes place in vivo is not known, but it is established that glyphosate depletes methionine by 50 to 65 percent in plants, as well as the aromatic amino acids (Nafziger *et al.*, 1984; Haderlie *et al.*, 1977). As we have already discussed, glyphosate interferes with cobalt bioavailability for cobalamin synthesis, and cobalamin is an essential catalyst for the conversion of cysteine to methionine.

Transglutaminase also cross-links proteins in the extracellular matrix, and therefore is important for wound healing, tissue remodeling, and stabilization of the extracellular matrix. Thus, autoimmunity to transglutaminase leads to destabilization of the microvilli lining the small intestines. Transglutaminase has 18 free cysteine residues which are targets for S-nitrosylation. A cysteine residue is also involved in the catalytic active site. A unique Ca^2+^ dependent mechanism regulates nitrosylation by NO, mediated by CysNO (S-nitrosocysteine). It was shown experimentally that up to 15 cysteines of transglutaminase were nitrosylated by CysNO in the presence of Ca^2+^, and this inhibited its enzymatic activity (Lai *et al.*, 2001).

Thus, another plausible mechanism by which glyphosate might enhance the development of autoantibodies to transglutaminase is by nitrosylating its cysteines, acting similarly to CysNO. A precedent for this idea is set with research proposing nitrosylation as the means by which glyphosate interferes with the heme active site in CYP enzymes (Lamb *et al.*, 1998). It is conceivable that cysteine nitrosylation by glyphosate at the active site inactivates the molecule, in which case glyphosate is itself acting as an “antibody.”

## 13 Evidence of glyphosate exposure in humans and animals

The US EPA has accepted Monsanto's claim that glyphosate is essentially harmless to humans. Due to this position, there have been virtually no studies undertaken in the US to assess glyphosate levels in human blood or urine. However, a recent study involving multiple countries in Europe provides disturbing confirmation that glyphosate residues are prevalent in the Western diet (Hoppe, 2013). This study involved exclusively city dwellers, who are unlikely to be exposed to glyphosate except through food sources. Despite Europe's more aggressive campaign against GMO foods than that in the Americas, 44% of the urine samples contained quantifiable amounts of glyphosate. Diet seems to be the main source of exposure. One can predict that, if a study were undertaken in the U.S., the percentage of the affected population would be much larger.

A recent study conducted on dairy cows in Denmark shows conclusively that the cows’ health is being adversely affected by glyphosate (Krüger *et al.*, 2013a). All of the cows had detectable levels of glyphosate in their urine, and it was estimated that from 0.1 to 0.3 mg of glyphosate was excreted daily from each cow. More importantly, all of the cows had serum levels of cobalt and manganese that were far below the minimum reference level for nutrient sufficiency. Half of the cows had high serum urea, and there was a positive linear relationship between serum urea and glyphosate excretion. High serum urea is indicative of nephrotoxicity. Blood serum levels of enzymes indicative of cytotoxicity such as creatine kinase (CK) and alkaline phosphatase (ALP) were also elevated. CK is indicative of rhabdomyolysis or kidney failure. High levels of ALP indicate liver damage, and it is often used to detect blocked bile ducts (Kaplan *et al.*, 1983).

Thus, the low cobalt levels and the indicators of liver, kidney, and gall bladder stress are all consistent with our previous discussion. The results of this study were also consistent with results of a study on rats exposed experimentally to glyphosate (Beuret *et al.*, 2005) in which Roundup was shown to be even more toxic than its active ingredient, glyphosate.

Glyphosate-metal complexes serve to reduce glyphosate's toxicity in the soil to plants, but they also protect glyphosate from attack by microorganisms that could decompose it (Cusiel, 2005). The degree of reactivity of the complex depends on which metals glyphosate binds to, which in turn depends upon the particular soil conditions (Nomura & Hilton, 1977). Glyphosate usually degrades relatively quickly (Vencill, 2002); however, a half-life of up to 22 years has also been reported in conditions where pH is low and organic matter contents are high (Nomura & Hilton, 1977). Therefore, glyphosate may survive much longer in certain soils than has been claimed by the industry, and could be taken up by crops planted subsequent to glyphosate application to kill weeds.

A disturbing trend of crop desiccation by glyphosate pre-harvest (O'Keeffe, 1980; O'Keeffe, 1981; Stride *et al.*, 1985; Darwent *et al.*, 1994; Orson & Davies, 2007) may be a key factor in the increased incidence of celiac disease. According to Monsanto, glyphosate was used on some 13% of the wheat area pre-harvest in the UK in 2004. However, by 2006 and 2007, some 94% of UK growers used glyphosate on at least 40% of cereal and 80% of oilseed crops for weed control or harvest management (Monsanto International Sàrl, 2010).

An increasing number of farmers now consider the benefits of desiccating their wheat and sugar cane crops with glyphosate shortly before the harvest (Monsanto International Sàrl, 2010). The advantage is improved harvesting efficiency because the quantity of materials other than grain or cane is reduced by 17%, due to a shutdown of growth following glyphosate treatment. Treated sugar cane crops produce drier stalks which can be baled more easily. There is a shorter delay before the next season's crop can be planted, because the herbicide was applied pre-harvest rather than post-harvest. Several pests can be controlled due to the fact that glyphosate is a broad-spectrum herbicide. These include Black grass, Brome grasses, and Rye grasses, and the suggestion is that this would minimize the risk of these weeds developing resistance to other herbicides.

A complete list of the latest EPA residue levels for glyphosate as of September 18, 2013 are shown in Table 1. Tolerances are established on all crops for both human and animal consumption resulting from the application of glyphosate.

**Table 1:** Complete list of glyphosate tolerances for residues in food crops in the U.S. as of September 18, 2013, as reported in: EPA: Title 40: Protection of Environment.

Commodity	PPM
Acerola	0.2
Alfalfa, seed	0.5
Almond, hulls	25
Aloe vera	0.5
Ambarella	0.2
Animal feed, nongrass, group 18	400
Artichoke, globe	0.2
Asparagus	0.5
Atemoya	0.2
Avocado	0.2
Bamboo, shoots	0.2
Banana	0.2
Barley, bran	30
Beet, sugar, dried pulp	25
Beet, sugar, roots	10
Beet, sugar, tops	10
Berry and small fruit, group 13-07	0.20
Betelnut	1.0
Biriba	0.2
Blimbe	0.2
Breadfruit	0.2
Cacao bean, bean	0.2
Cactus, fruit	0.5
Cactus, pads	0.5
Canistel	0.2
Canola. seed	20
Carrot	5.0
Chaya	1.0
Cherimoya	0.2
Citrus, dried pulp	1.0
Coconut	0.1
Coffee, bean, green	1.0
Corn, pop, grain	0.1
Corn, sweet, kernel plus cob with husk removed	3.5
Cotton, gin byproducts	210
Custard apple	0.2
Dried fruit	0.2
Dokudami	2.0
Durian	0.2
Epazote	1.3
Feijoa	0.2
Fig	0.2
Fish	0.25
Fruit, citrus, group 10-10	0.50
Fruit, pome, group 11-10	0.20
Fruit, stone, group 12	0.2
Galangal, roots	0.2
Ginger, white, flower	0.2
Gourd, buffalo, seed	0.1
Governor's plum	0.2
Gow kee, leaves	0.2
Grain, cereal, forage, fodder and straw, group 16, except field corn, forage and field corn, stover	100
Grain, cereal, group 15 except field corn, popcorn, rice, sweet corn, and wild rice	30
Grass, forage, fodder and hay, group 17	300
Guava	0.2
Herbs subgroup 19A	0.2
Hop, dried cones	7.0
llama	0.2
Imbe	0.2
Imbu	0.2
Jaboticaba	0.2
Jackfruit	0.2
Kava, roots	0.2
Kenaf, forage	200
Leucaena, forage	200
Longan	0.2
Lychee	0.2
Mamey apple	0.2
Mango	0.2
Mangosteen	0.2
Marmalade box	0.2
Mioga, flower	0.2
Noni	0.20
Nut, pine	1.0
Nut, tree, group 14	1.0
Oilseeds, group 20, except canola	40
Okra	0.5
Olive	0.2
Oregano, Mexican, leaves	2,0
Palm heart	0.2
Palm heart, leaves	0.2
Palm, oil	0.1
Papaya	0.2
Papaya, mountain	0.2
Passionfruit	0.2
Pawpaw	0.2
Pea, dry	8.0
Peanut	0.1
Peanut, hay	0.5
Pepper leaf, fresh leaves	0.2
Peppermint, tops	200
Perilla, tops	1.8
Persimmon	0.2
Pineapple	0.1
Pistachio	1.0
Pomegranate	0.2
Pulasan	0.2
Quinoa. grain	5.0
Rambutan	0.2
Rice, grain	0.1
Rice, wild, grain	0.1
Rose apple	0.2
S apod ilia	0.2
Sapote, black	0.2
Sapote, mamey	0.2
Sapote, white	0.2
Shellfish	3.0
Soursop	0.2
Spanish lime	0.2
Spearmint, tops	200
Spice subgroup 19B	7.0
Star apple	0.2
Starfruit	0.2
Stevia, dried leaves	1.0
Sugar apple	0.2
Sugarcane, cane	2.0
Sugarcane, molasses	30
Surinam cherry	0.2
Sweet potato	3.0
Tamarind	0.2
Tea. dried	1.0
Tea, instant	7.0
Teff, forage	100
TefF, grain	5.0
Teff, hay	100
Ti, leaves	0.2
Ti, roots	0.2
Ugli fruit	0.5
Vegetable, bulb, group 3-07	0.20
Vegetable, cucurbit, group 9	0.5
Vegetable, foliage of legume, subgroup 7A, except soybean	0.2
Vegetable, fruiting, group 8-10 (except okra)	0.10
Vegetable, leafy, brassica. group 5	0.2
Vegetable, leafy, except brassica, group 4	0.2
Vegetable, leaves of root and tuber, group 2, except sugar beet tops	0.2
Vegetable, legume, group 6 except soybean and dry pea	5.0
Vegetables, root and tuber, group 1, except carrot, sweet potato, and sugar beet	0.20
Wasabi. roots	0.2
Water spinach, tops	0.2
Watercress, upland	0.2
Wax jambu	0.2

As glyphosate usage continues unabated, glyphosate resistance among weeds is becoming a growing problem (Waltz, 2010), necessitating a strategy that either involves an increase in the amount of glyphosate that is applied or a supplementation with other herbicides such as glufosinate, dicampa, 2-4D, or atrazine. Agrochemical companies are now actively developing crops with resistance to multiple herbicides (Culpepper, 2000), a disturbing trend, especially since glyphosate's disruption of CYP enzymes leads to an impaired ability to break down many other environmental chemicals in the liver.

## 14 Kidney disease in agricultural workers

**Figure 4:**
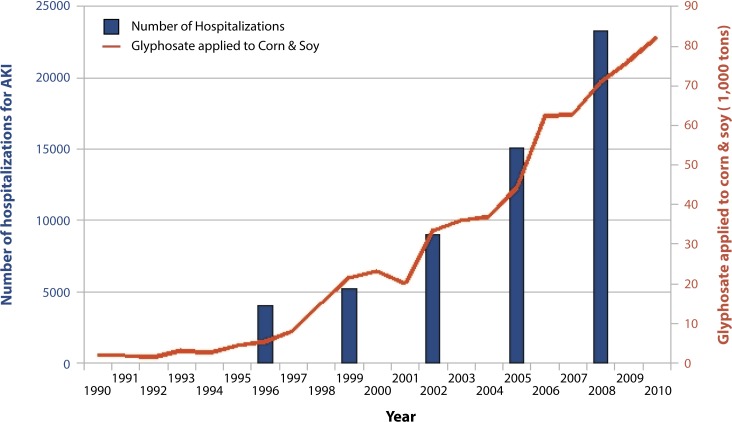
Number of hospitalizations for acute kidney injury plotted against glyphosate applied to com & soy (in 1000 tons). (Figure courtesy of Nancy Swanson).

**Figure 5:**
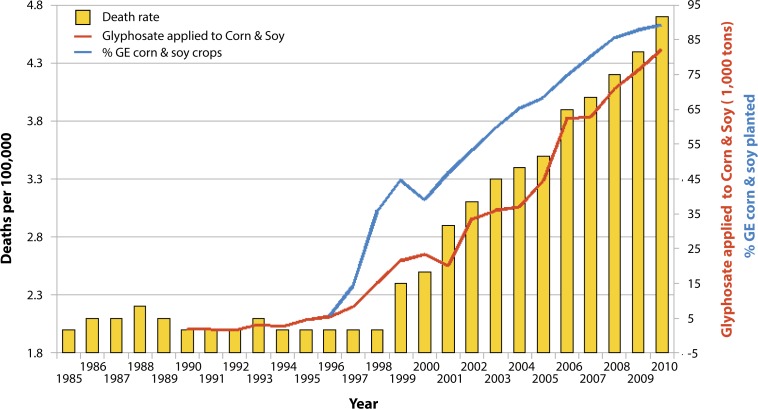
End stage renal disease deaths (ICD N18.0 & 585.6) plotted against % GE corn & soy planted (R=0.9585, p<4.03e-6) and glyphosate applied to corn & soy (R=0.9844, *p≤*3.704e-09). Sources: USDA:NASS; CDC. (Figure courtesy of Nancy Swanson).

Chronic kidney disease is a globally increasing problem (Ramirez-Rubio *et al.*, 2013), and glyphosate may be playing a role in this epidemic. A plot showing recent trends in hospitalization for acute kidney injury aligned with glyphosate usage rates on corn and soy shows strong correlation, as illustrated in Figure 4, and a similar correlation is seen for deaths due to end-stage renal disease in Figure 5. Recently, it has been noted that young men in Central America are succumbing in increasing numbers to chronic kidney disease (Trabanino *et al.*, 2002; Cerdas, 2005; Torres *et al.*, 2010; Peraza *et al.*, 2012; Ramirez-Rubio *et al.*, 2013; Sanoff *et al.*, 2010). The problem appears to be especially acute among agricultural workers, mainly in sugar cane fields (Cerdas, 2005; Torres *et al.*, 2010; Peraza *et al.*, 2012). Since we have shown in Section 8 how glyphosate can produce toxic effects on the kidneys through its disruption of gut bacteria, it is fruitful to consider whether glyphosate could be playing a role in the fate of Central American workers in the sugar cane fields.

In attempting to explain this phenomenon, physicians and pharmacists have proposed that it may be due to dehydration caused by over-exertion in high temperature conditions, combined with an acute reaction to commonly administered non-steroidal anti-inflammatory drugs (NSAIDs) to treat pain and/or antibiotics to treat infection (Ramirez-Rubio *et al.*, 2013). NSAIDs require CYP enzymes in the liver for detoxification (Agúndez *et al.*, 2009), so impaired CYP function by glyphosate would lead to a far more toxic effect of excessive NSAID administration. Kidney disease among agricultural workers tends to be associated with chronic glomerulonephritis and interstial nephritis, which was proposed in (Soderland *et al.*, 2010) to be due to environmental toxins such as heavy metals or toxic chemicals. Glomerulonephritis is also found in association with celiac disease (Katz *et al.*, 1979; Peters *et al.*, 2003). A Swedish study showed a five-fold increase in nephritis risk in celiac patients (Peters *et al.*, 2003).

A strong hint comes from epidemiological studies conducted in Costa Rica (Cerdas, 2005). The demographic features of those with chronic renal failure revealed a remarkably specific pattern of young men, between 20 and 40 years old, with chronic interstitial nephritis. All of them were sugar-cane workers. These authors wrote: ”A specific study of their work environment is needed to determine what in their daily activities puts them at increased risk for chronic renal failure.”

Agriculture is an important part of the economy of the state of Louisiana in the United States, and sugar cane is a significant agricultural product. Chemical methods to ripen sugar cane are commonly used, because they can substantially increase the sucrose content of the harvest (Richard & Dalley, 2009). Glyphosate, in particular, has been the primary ripener used in Louisiana since 1980 (Orgeron, 2012). As of 2001, Louisiana had the highest rate of kidney failure in the U.S. (State-Specific Trends in Chronic Kidney Failure – United States, 1990–2001). Louisiana's death rate per 100,000 from nephritis/kidney disease is 26.34 as compared to a U.S. rate of 14.55 (Network Coordinating Council, 2013). The number of patients on dialysis has risen sharply in the last few years.

By 2005, it is estimated that 62% of the total harvested hectares of sugar cane in Louisiana were ripened with glyphosate (Legendre *et al.*, 2005). A paper published in 1990 showed that glyphosate applied as a ripener on three different sugar cane varieties grown *in Costa Rica* produced up to a 15% increase in the sucrose content of the harvested sugar cane (Subiros, 1990). Glyphosate applied before the harvest is the *only* sugarcane ripener currently registered for use in the U.S.

A disturbing recent trend is the repeated application of glyphosate over the course of the season with the hope of further increasing yields (Richard & Dalley, 2009). Responses to the standard application rate (0.188 lb/acre) of glyphosate have been inconsistent, and so farmers are increasing both the amount and the frequency of application. In (Richard & Dalley, 2009), growers are encouraged not to apply glyphosate beyond mid-October, as results are counterproductive, and not to use higher rates in an attempt to improve yield. But it is doubtful that these recommendations are being followed. It is likely, although we have not been able to confirm this, that glyphosate usage has expanded in scope on the sugar cane fields in Central America since 2000, when the expiration of Monsanto's patent drove prices down, and that the practices of multiple applications of glyphosate in the U.S. are also being followed in Central America. Several other ripening agents exist, such as Ethephon, Trinexapacethyl, and Sulfometuron-methyl, but glyphosate is likely growing in popularity recently due to its more favorable pricing and perceived non-toxicity. Larger amounts are needed for effective ripening in regions that are hot and rainy, which matches the climate of Costa Rica and Nicaragua.

## 15 Discussion

In this paper, we have developed an argument that the alarming rise in the incidence of celiac disease in the United States and elsewhere in recent years is due to an increased burden of herbicides, particularly glyphosate exposure in the diet. We suggest that a principal factor is the use of glyphosate to desiccate wheat and other crops prior to the harvest, resulting in crop residue and increased exposure. Strong evidence for a link between glyphosate and celiac disease comes from a study on predatory fish, which showed remarkable effects in the gut that parallel the features of celiac disease (Shenapati *et al.*, 2009).

More generally, inflammatory bowel disease has been linked to several environmental factors, including a higher socioeconomic status, urban as opposed to rural dwelling, and a “Westernized” cultural context (Shapira *et al.*, 2010). Disease incidence is highest in North America and Europe, and is higher in northern latitudes than in southern latitudes within these regions, suggesting a beneficial role for sunlight. According to the most recent statistics from the U.S. Environmental Protection Agency (EPA) (Grube *et al.*, 2011), the U.S. currently represents 25% of the total world market on herbicide usage. Glyphosate has been the most popular herbicide in the U.S. since 2001, whereas it was the 17th most popular herbicide in 1987 (Kiely *et al.*, 2004). Since 2001, glyphosate usage has grown considerably, due to increased dosing of glyphosate-resistant weeds and in conjunction with the widespread adoption of “Roundup-Ready” genetically modified crops. Glyphosate is probably now the most popular herbicide in Europe as well (Kimmel *et al.*, 2013). Glyphosate has become the number one herbicide worldwide, due to its perceived lack of toxicity and its lower price after having become generic in 2000 (Duke & Powles, 2008).

A recent estimate suggests that one in twenty people in North America and Western Europe suffer from celiac disease (Koning, 2005; Fasano *et al.*, 2003). Outdoor occupational status is protective (Sonnenberg *et al.*, 1991). First generation immigrants into Europe or North America are generally less susceptible, although second generation non-Caucasian immigrants statistically become even more susceptible than native Caucasians (Shapira *et al.*, 2010). This may in part stem from the increased need for sunlight exposure given darker skin pigmentation.

Table 2 summarizes our findings relating glyphosate to celiac disease. All of the known biological effects of glyphosate – cytochrome P450 inhibition, disruption of synthesis of aromatic amino acids, chelation of transition metals, and antibacterial action – contribute to the pathology of celiac disease.

**Table 2:** Illustration of the myriad ways in which glyphosate can be linked to celiac disease or its associated pathologies. (a) Disruption of gut bacteria

Glyphosate Effect	Dysfunction	Consequences
reduced Bifidobacteria	impaired gluten breakdown	transglutaminase antibodies
reduced Lactobacillus	impaired phytase breakdown reduced selenoproteins	metal chelation autoimmune thyroid disease
anaerobic E. coli	indole toxicity	kidney failure
C. diff overgrowth	p-Cresol toxicity	kidney failure
Desulfovibrio overgrowth	hydrogen sulfide gas	inflammation

**Table:** (b) Transition metal chelation

Glyphosate Effect	Dysfunction	Consequences
cobalt deficiency	cobalamin deficiency reduced methionine elevated homocysteine	neurodegenerative diseases impaired protein synthesis heart disease
molybdenum deficiency	inhibited sulfite oxidase inhibited xanthine oxidase	impaired sulfate supply DNA damage/cancer teratogenesis megaloblastic anemia
iron deficiency		anemia

**Table:** (c) CYP enzyme inhibition

Glyphosate Impairment	Dysfunction	Consequences
vitamin D3 inactivation	impaired calcium metabolism	osteoporosis; cancer risk
retinoic acid catabolism	suppressed transglutaminase	teratogenesis
bile acid synthesis	impaired fat metabolism impaired sulfate supply	gall bladder disease pancreatitis
xenobiotic detoxification	increased toxin sensitivity impaired indole breakdown	liver disease macrocytic anemia kidney failure
nitrate reductase	venous constriction	venous thrombosis

**Table:** (d) Shikimate pathway suppression

Glyphosate Effect	Dysfunction	Consequences
tryptophan deficiency	impaired serotonin supply hypersensitive receptors	depression nausea, diarrhea

Celiac disease is associated with deficiencies in several essential micronutrients such as vitamin D3, cobalamin, iron, molybdenum, selenium and the amino acids, methionine and tryptophan, all of which can be explained by glyphosate. Glyphosate depletes multiple minerals in both genetically modified soybeans (Saes *et al.*, 2010) and conventional soybeans (Cakmak *et al.*, 2009), which would translate into nutritional deficiencies in foods derived from these crops. This, together with further chelation in the gut by any direct glyphosate exposure, could explain deficiencies in cobalt, molybdenum and iron. Glyphosate's effect on CYP enzymes should lead to inadequate vitamin D3 activation in the liver (Hietanen *et al.*, 1983; Ponchon *et al.*, 1969). Cobalamin depends on cobalt, and cobalt-dependent enzymes in plants and microbes have been shown to be inhibited by glyphosate (Bode *et al.*, 1984; Ganson and Jensen, 1988). Glyphosate has been shown to severely impair methionine and tryptophan synthesis in plants (Nafziger *et al.*, 1984), which would reduce the bioavailability of these nutrients in derived foods.

There are multiple intriguing connections between celiac disease and microcephaly, all of which can be linked to glyphosate. Celiac disease is found in association with microcephaly in infants (Bostwick *et al.*, 2001; Lapunzina, 2002), and teratogenic effects are also observed in children born to celiac mothers (Dickey *et al.*, 1996; Martinelli *et al.*, 2000). Microcephaly in an infant where confirmed molybdenum deficiency was present (Boles *et al.*, 1993) suggests that molybdenum deficiency could be causal. However, elevated RA also induces microcephaly, as does indole-3-acetic acid, which has been dramatically linked to microcephaly in mice (Furukawa *et al.*, 2007). Elevated RA is predicted as a response to glyphosate due to its expected inhibition of CYP enzymes which catabolize RA in the liver (Lamb *et al.*, 1998; Hietanen *et al.*, 1983). Molybdenum deficiency is expected due to glyphosate's ability to chelate cationic minerals. Glyphosate has been shown to induce indole-3-acetic acid synthesis in plants (Caal *et al.*, 1987), and it induces a shift to anaerobic metabolism in E. coli (Lu *et al.*, 2013), which is associated with indole synthesis.

Celiac disease is associated with impaired serotonin metabolism and signaling in the gut, and this feature leads us to propose a novel role for serotonin in transporting sulfate to the tissues. It is a curious and little known fact that glucose and galactose, but not fructose or mannose, stimulate 5-HT synthesis by EC cells in the intestinal lumen (Kim *et al.*, 2001), suggesting a role for EC cells as “glucose sensors.” Glucose and galactose are the two sugars that make up the heparan sulfate chains of the syndecans and glypicans that attach to the membrane-bound proteins in most cells, serving as the innermost constituency of the extracellular matrix (Bernfield *et al.*, 1999). In (Seneff *et al.*, 2012), it was proposed that part of the post-prandial glucose that is taken up by the tissues is temporarily stored in the extracellular matrix as heparan sulfate, and that a deficiency in sulfate supply impairs this process, which impedes glucose uptake in cells. These heparan sulfate units have a high turnover rate, as they are typically broken down within three hours of their initial placement (Turnbull *et al.*, 2001). This provides the cells with a convenient temporary buffer for glucose and galactose that can allow them to more efficiently remove these sugars from the serum. Insufficient sulfate supplies would impair this process and lead to insulin resistance.

As is the case for other monoamine neurotransmitters as well as most sterols, 5-HT is normally transported in the serum in a sulfated form. The sulfate moiety must be removed for the molecule to activate it. Therefore, 5-HT, as well as these other monoamine neurotransmitters and sterols, can be viewed as a sulfate “escort” in the plasma. In (Samsel & Seneff, 2013), it was argued that such carbon-ring-containing molecules are necessary for safe sulfate transport, especially in the face of co-present kosmotropes like glyphosate, in order to protect the blood from excess viscosity during transport. Support for the concept that glyphosate gels the blood comes from the observation that disseminated coagulation is a characteristic feature of glyphosate poisoning (Zouaoui *et al.*, 2013). Since glyphosate disrupts sterol sulfation and it disrupts monoamine neurotransmitter synthesis, in addition to its physical kosmotropic feature, it can be anticipated that a chronic exposure to even a small amount of glyphosate over the course of time will lead to a system-wide deficiency in the supply of sulfate to the tissues. We believe that this is the most important consequence of glyphosate's insidious slow erosion of health.

An interesting consideration regarding a known link between celiac disease and hypothyroidism (Collins *et al.*, 2012) emerges when one considers that iodide is one of the few chaotropic (structure breaking) anions available to biological systems: another important one being nitrate, which is elevated in the urine in association with celiac disease (Laurin *et al.*, 2003). It is intriguing that the conversion of T4 to T3 (the active form of thyroid hormone) involves selenium as an essential cofactor. Furthermore, iodide is released in the process, thus providing chaotropic buffering in the blood serum. Therefore, impaired conversion due to deficient selenium results in an inability to buffer this significant chaotrope in the blood, despite the fact that chaotropic buffering is likely desperately needed in the context of the kosmotropic effects of glyphosate. While speculative, it is possible that the autoimmune thyroid disease that develops in association with celiac disease is a direct consequence of the inability to activate thyroid hormone due to insufficient selenium. Indeed, celiac patients with concurrent hypothyroidism require an elevated dose of levothyroxine (T4) compared to non-celiac hypothyroid patients (Collins *et al.*, 2012), which could be due to impaired activation to T3.

The link between autoimmune (type 1) diabetes and autoimmune thyroiditis is likely tied to deficiencies in selenoproteins leading to apoptosis. Diabetic rats produce significantly less glomerular heparan sulfate in the kidneys than controls, and this is associated with increased albuminurea (Jaya *et al.*, 1993). However, children with type-1 diabetes and celiac disease excrete lower levels of albumin than type-1 diabetic children without celiac disease, suggesting a protective role for celiac disease (Gopee *et al.*, 2013). Wheat is a good source of tryptophan, so it is likely that tryptophan-derived serotonin induces the symptoms of diarrhea and nausea associated with wheat ingestion, but, at the same time, transports available sulfate through the vasculature, to help maintain adequate supplies of heparan sulfate to the glomerulus. Thus, the increased metabolism of dietary tryptophan to serotonin observed in association with celiac disease may help ameliorate the sulfate deficiency problem. Glyphosate's interference with CYP enzymes links to impaired bile-acid production in the liver, which in turn impairs *sterol*-based sulfate transport, placing a higher burden on serotonin for this task.

We have argued here that kidney failure, a known risk factor in celiac disease, is a consequence of depleted sulfate supplies to the kidneys. An alarming increase in kidney failure in young male agricultural workers in sugar cane fields in South America can be directly linked to the recent increase in the practice of using Roundup to “ripen” the crop just prior to the harvest. Furthermore, glyphosate's interference with selenoprotein supply would lead to thyroid dysfunction, which greatly increases risk to kidney disease. We propose here that glyphosate is the key environmental factor contributing to this epidemic, but further investigation is warranted.

**Figure 6:**
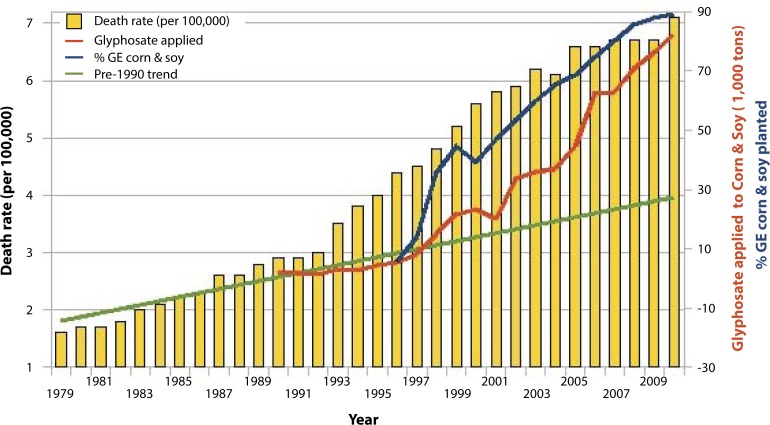
Deaths from Parkinson's disease (ICD G20 & 332.0) plotted against glyphosate use on corn & soy (R=0.9006, *p≤*5.063e-07) and % GE corn & soy planted (R=0.9676, *p≤*2.714e-06). Sources: USDA:NASS; CDC. (Figure courtesy of Nancy Swanson).

While we have covered a broad range of pathologies related to celiac disease in this paper, and have shown how they can be explained by glyphosate exposure, there are likely still other aspects of the disease and the connection to glyphosate that we have omitted. For example, in a remarkable case study (Barbosa, 2001), a 54-year-old man who accidentally sprayed himself with glyphosate developed skin lesions six hours later. More significantly, one month later he exhibited symptoms of Parkinson's disease. Movement disorders such as Parkinsonism are associated with gluten intolerance (Baizabal-Carvallo, 2012). Figure 6 shows plots of glyphosate application to corn and soy alongside plots of deaths due to Parkinson's disease. These and other connections will be further explored in future research.

## 16 Conclusion

Celiac disease is a complex and multifactorial condition associated with gluten intolerance and a higher risk to thyroid disease, cancer and kidney disease, and there is also an increased risk to infertility and birth defects in children born to celiac mothers. While the principal diagnostic is autoantibodies to tissue transglutaminase, celiac disease is associated with a spectrum of other pathologies such as deficiencies in iron, vitamin D3, molybdenum, selenium, and cobalamin, an overgrowth of pathogens in the gut at the expense of beneficial biota, impaired serotonin signaling, and increased synthesis of toxic metabolites like p-Cresol and indole-3-acetic acid. In this paper, we have systematically shown how all of these features of celiac disease can be explained by glyphosate's known properties. These include (1) disrupting the shikimate pathway, (2) altering the balance between pathogens and beneficial biota in the gut, (3) chelating transition metals, as well as sulfur and selenium, and (4) inhibiting cytochrome P450 enzymes. We argue that a key system-wide pathology in celiac disease is impaired sulfate supply to the tissues, and that this is also a key component of glyphosate's toxicity to humans.

The monitoring of glyphosate levels in food and in human urine and blood has been inadequate. The common practice of desiccation and/or ripening with glyphosate right before the harvest ensures that glyphosate residues are present in our food supply. It is plausible that the recent sharp increase of kidney failure in agricultural workers is tied to glyphosate exposure. We urge governments globally to reexamine their policy towards glyphosate and to introduce new legislation that would restrict its usage.

## Notes

***Disclosures:** The authors have nothing to disclose.*
